# Thromboembolic Complications After Bariatric Surgery: Is the High Risk Real?

**DOI:** 10.7759/cureus.33444

**Published:** 2023-01-06

**Authors:** Lucia Carvalho, Rui F Almeida, Mário Nora, Marta Guimarães

**Affiliations:** 1 General Surgery, Centro Hospitalar de Entre Douro e Vouga, Santa Maria da Feira, PRT

**Keywords:** thromboprophylaxis protocol, bariatric protocol, thromboembolic events, thromboprophylaxis, bariatric surgery

## Abstract

Introduction: Nowadays, a large number of bariatric surgery (BS) procedures are undertaken worldwide as surgery has become an efficient strategy to treat the obesity epidemic. The risk of venous thromboembolism (VTE) is increased in patients undergoing BS not only due to the intrinsic surgical risk but also because patients with obesity have a 2-3-fold higher risk of VTE. The optimal strategy for VTE prevention in BS setting, including optimal dose and thromboprophylaxis regimen, is still not fully clarified. The aim of this study was to report a bariatric high-volume center experience and to propose a practical thromboprophylaxis protocol for this population.

Methods: A single-center, observational, retrospective, and longitudinal study was conducted from January 2018 to December 2020, a total of 901 patients who underwent primary and revisional bariatric surgery were included.

Results: The overall frequency of VTE events was 0.44% (n=4), one patient had pulmonary embolism (PE) during index hospital admission; another patient had simultaneous deep venous thrombosis (DVT) and PE, two months after surgery; and two other patients had DVT, nine and 16 months after surgery. The median time for VTE was four months. The incidence of females was 75% (n=3) and the median age was 57 years. Only one female patient was using oral contraception. None had a previous history of thromboembolic events, chronic venous insufficiency, or other known diseases that could increase the VTE risk.

Conclusion: Considering the outcomes reported by this experienced center with low rates of thromboembolic events, we suggest a thromboprophylaxis protocol that can be easily applied to the majority of bariatric patients.

## Introduction

Over the past decades, the number of successful bariatric surgeries has increased with excellent results on weight loss and obesity-related morbidity. Nevertheless, bariatric surgery (BS) is a technically challenging procedure performed on high-risk patients which can be associated with potential medical and surgical complications such as venous thromboembolic (VTE) events [1,2]. VTE is an uncommon complication following BS but a leading cause of significant postoperative morbidity and mortality. The rate of venous thromboembolic events following bariatric procedures, comprising deep vein thrombosis (DVT), pulmonary embolism (PE), and porto-spleno-mesenteric (PSM) venous thrombosis, is 0.3-2.4% [3-5], despite preventive methods [6]. PE, one of the leading causes of mortality after BS, has 1% incidence but counts for 40% of all deaths within 30 days postoperatively [7-10].

Bariatric surgery patients have a two-fold increased risk for VTE events. Obesity is itself a hypercoagulability state, frequently associated with hypertension (HTN), obstructive sleep apnea (OSA), and venous stasis, which are known risk factors for VTE-related complications. On the other hand, bariatric surgery comprises a range of complex procedures. The extended operative time, the occurrence of surgical complications, the need for surgical reinterventions, and even blood transfusions are risk factors for VTE events [11].

Various strategies have been used to prevent VTE in bariatric surgery patients, including pharmacologic and mechanical thromboprophylaxis. However, the optimal strategy, including thromboprophylaxis regimen, remains uncertain.

The aim of this paper was to report the experience of a high-volume bariatric center in the prevention of venous thromboembolic events and to propose a standard thromboprophylaxis protocol for this specific population undergoing bariatric surgery.

## Materials and methods

Study population

A retrospective study was conducted including all patients admitted to BS from January 2018 to December 2020 in a single surgical center. After ethics committee approval, baseline demographic characteristics, preoperative risk factors for VTE events, surgical details, perioperative outcomes (such as thromboembolic and hemorrhagic events), complications management, and mortality data were collected from electronic medical records.

Patients were evaluated in a multidisciplinary setting composed of bariatric surgeon, anesthesiologist, endocrinologist, psychologist, and nutritionist prior to being indicated for bariatric surgery according to the International Federation for the Surgery of Obesity and Metabolic Disorders (IFSO) criteria. The surgical technique for each individual patient was selected based on age, body mass index (BMI), presence of gastroesophageal reflux symptoms, and metabolic comorbidities. The *Helicobacter pylori *status, familiar history of gastric cancer, and gastric lesions requiring endoscopic follow-up are also factors considered during surgical procedure choice. The diagnosis of VTE events was searched in the presence of clinical symptoms or signs. The diagnosis of DVT was made with venous ultrasound and PE by computed tomography pulmonary angiography.

Ethical approval for this study was obtained from the hospital ethics committee (ID: CE 55-2022). Informed consent was obtained from all individual participants included in the study.

Venous thromboembolism prophylaxis protocol

Routine thromboprophylaxis for patients with obesity without any other additional risk factors includes subcutaneous injection of low molecular weight heparin (LMWH) - enoxaparin, 40 mg/day, starting 12 h after surgery (Table 1). In patients with additional risk factors, thromboprophylaxis included LMWH and mechanical methods, such as graduated compression stockings or pneumatic compression devices during surgery. Only patients with a BMI over 50 or weight over 150 kg, receive an adjusted LMWH dose of 60 mg/day. Chemical thromboprophylaxis is not routinely administered before surgery. Postoperatively early ambulation 6 h after surgery is recommended and an extended course of LMWH for three weeks postoperatively is indicated in all patients.

**Table 1:** Thromboprophylaxis protocol for patients who underwent bariatric surgery. CPAP: continuous positive airway pressure; BiPAP: bilevel positive airway pressure Higher-risk patients requiring ICU admission - patients with BMI >50 kg/m^2^ or with major cardiovascular, pulmonary, and/or neuromuscular diseases.

Mechanical prophylaxis
a. Graduated compression stockings (GCS)
b. Dynamical system using intermittent pneumatic compression (IPC) in higher-risk patients requiring ICU admission.
Chemical prophylaxis
a. Enoxaparin 40 mg per day starting 12 h after surgery and continuing for 3 weeks.
b. Enoxaparin 60 mg per day in patients with BMI >50 kg/m^2^ or weight >150 kg.
Postoperative care
a. Non-invasive ventilation (CPAP, BiPAP, or high flow cannula) in immediate postoperative care.
b. Early mobilization - ambulation on the day of surgery.
c. Start liquids 6 h after surgery, as tolerated.
d. No tubes - no nasogastric tube, no urinary catheter, no abdominal drains.
e. Non-opioid based analgesia.
f. Early discharge.

Statistical analysis 

Statistical analyses were carried out using IBM SPSS Statistics for Windows, version 26.0 (Armonk, NY: IBM Corp.). Data are presented as mean±standard deviation (SD) or median (interquartile range Q1-Q3) according to normal distribution of the continuous variables. Categorical variables are expressed as frequency percentages. The Mann-Whitney U, Pearson chi-square, and Fisher’s exact test were used as appropriate. A two-sided p-value of <0.05 was considered statistically significant.

## Results

Data from patients submitted to bariatric procedures between January 2018 and December 2020 were collected, with follow-up ranging from 20 to 54 months. During these 36 months, 901 patients underwent primary and revisional bariatric surgery with a post-procedure multidisciplinary follow-up, starting three days after discharge. The median age of the cohort was 44 years and 716 patients (79.5%) were female. The median BMI was 41 kg/m^2^ (Table 2).

**Table 2:** Patients' demographic and anthropometric data. Follow-up duration and surgeries performed per year. IQR: interquartile range; BMI: body mass index

Demographic		N=901
Age, years (IQR)		44 (35-51)
Female, n (%)		716 (79.5%)
BMI, kg/m^2^ (IQR)		41 (38-44.7)
Follow-up time, months (IQR)		34 (29-42)
Surgeries per year, n (%)	2018	208 (23.1%)
	2019	412 (45.7%)
	2020	281 (31.2%)

The study population was evaluated according to past medical history, which included risk factors for VTE events. The most common risk factors were peripheral venous disease (15.3%), hormonal therapy (39.8% of female patients), past and present smoking habits (28.9%), and pulmonary diseases (21.1%) (Table 3).

**Table 3:** Patient’s preoperative risk factors for venous thromboembolism. VTE: venous thromboembolism; IUDS: intrauterine device; OSA: obstructive sleep apnea

Variables	Values
Previous VTE, n (%)	7 (0.8%)
Peripheral venous disease, n (%)	138 (15.3%)
Thrombophilia, n (%)	2 (0.2%)
Hormonal therapy, n (%)	285 (39.8% of female patients)
a. Combined oral contraceptive	165 (23%)
b. Combined transdermal patch	2 (0.3%)
c. Combined vaginal ring	14 (2%)
d. Progestogen-only oral contraceptive	26 (3.6%)
e. Progestogen-only implants	17 (2.4%)
f. Hormonal IUDs	56 (7.8%)
g. Copper IUDs	4 (0.6%)
h. Hormonal replacement therapy	1 (0.1%)
Smoking habits, n (%)	260 (28.9%)
a. Current smokers	139 (15.4%)
b. Former smokers	121 (13.4%)
Respiratory disease, n (%)	193 (21.1%)
a. OSA	130 (14.4%)
b. Obesity-hypoventilation syndrome	3 (0.3%)
c. Asthma	56 (6.2%)
d. Chronic obstructive pulmonary disease	4 (0.4%)
Cardiovascular risk factors, n (%)	
a. Arterial hypertension	347 (38.5%)
b. Diabetes mellitus	167 (18.5%)
c. Hyperlipidemia	263 (29.2%)
Inflammatory intestinal disease, n (%)	1 (0.1%)

The most commonly performed surgical technique was the Roux-en-Y gastric bypass (74%) followed by Single anastomosis duodeno-ileal bypass with sleeve gastrectomy (11.9%) and Sleeve gastrectomy (7.2%). Revisional techniques comprised 4.8% of all procedures. All surgeries were laparoscopic with no conversion to laparotomy observed (Table 4).

**Table 4:** Bariatric surgical procedures and operative data. IQR: interquartile range; AGB: adjustable gastric band; OAGB: one anastomosis gastric bypass; SG: sleeve gastrectomy; RYGB: Roux-en-Y gastric bypass; S-RYGB: standard Roux-en-Y gastric bypass; M-RYGB: metabolic Roux-en-Y gastric bypass; SADI-S: single anastomosis duodeno-ileal bypass with sleeve gastrectomy; DS: duodenal switch Standard RYGB and metabolic RYGB differ in lengths of biliopancreatic limb length as previous reported [12].

Procedures	N=901
Laparoscopic approach, n (%)	901 (100%)
Operative time, min (IQR)	60 (45-70)
Techniques - primary surgery, n (%)	858 (95.2%)
AGB removal	5 (0.6%)
SG	65 (7.2%)
a. SG	64 (7.1%)
b. SG+cholecystectomy	1 (0.1%)
OAGB	1 (0.1%)
S-RYGB	573 (63.6%)
a. S-RYGB	534 (59.3%)
b. S-RYGB+cholecystectomy	5 (0.6%)
c. S-RYGB with gastrectomy of excluded stomach	32 (3.6%)
d. S-RYGB with gastrectomy of excluded stomach and cholecystectomy	2 (0.2%)
M-RYGB	94 (10.4%)
a. M-RYGB	93 (10.3%)
b. M-RYGB with left adrenalectomy	1 (0.1%)
SADI-S	107 (11.9%)
a. SADI-S	106 (11.8%)
b. SADI-S with cholecystectomy	1 (0.1%)
DS	13 (1.4%)
Techniques - revisional surgery, n (%)	43 (4.8%)
AGB	
a. Conversion to SG	1 (0.1%)
b. Conversion to S-RYGB	16 (1.8%)
c. Conversion to M-RYGB	1 (0.1%)
Gastroplication	
a. Conversion to S-RYGB	1 (0.1%)
b. Conversion to S-RYGB with gastrectomy of excluded stomach	1 (0.1%)
SG	
a. Conversion to S-RYGB	1 (0.1%)
b. Conversion to SADI-S	2 (0.2%)
c. Conversion to DS	4 (0.4%)
RYGB	
a. Limb distalization	10 (1.1%)
b. Conversion to SADI-S	1 (0.1%)
c. Conversion to DS	1 (0.1%)
SADI-S	
a. Conversion to S-RYGB	1 (0.1%)
b. Conversion to DS	1 (0.1%)
Revision of Nissen fundoplication to M-RYGB	1 (0.1%)
Revision of Nissen fundoplication to SADI-S	1 (0.1%)

Median follow-up time was 34 months. During this period, only four patients experienced VTE events: one PE during index hospital admission, one simultaneous DVT and PE 2 months after surgery, and two DVT, 9 and 16 months after surgery. Overall frequency of VTE events was 0.44% (n=4), averaging 4 months after BS and 75% of post-BS VTE events occurred after hospital discharge. There were no statistically significant differences observed in age, gender, BMI and comorbidities when comparing with the group without VTE. The frequency of males among VTE patients was 25% (n=1). The 53-year-old male patient was a smoker with metabolic syndrome and OSA. Regarding female patients, only one was using oral contraception (Table 5).

**Table 5:** Characteristics of the patients with VTE events. *Mann-Whitney U test. **Pearson chi-square. ***Fisher’s exact test. IQR: interquartile range; BMI: body mass index; VTE: venous thromboembolic; DVT: deep venous thrombosis; PE: pulmonary embolism; S-RYGB: standard Roux-en-Y gastric bypass; M-RYGB: metabolic Roux-en-Y gastric bypass; SADI-S: single anastomosis duodeno-ileal bypass with sleeve gastrectomy; OSA: obstructive sleep apnea

Variables		n = 4 (0.44%)	p-Value
Age, years (IQR)		57 (41.5-59.8)	0.08*
Female, n (%)		3 (75%)	0.974**
Preoperative BMI, kg/m^2^ (IQR)		42.5 (35-45)	0.985*
Postdischarge VTE events, n (%)		3 (0.75%)	-
Time after surgery, months (IQR)		4 (2-9)	-
Type of VTE event, n (%)	DVT	2 (0.22%)	-
	PE	1 (0.11%)	
	DVT+PE	1 (0.11%)	
Procedure, n (%)	S-RYGB	1 (0.11%)	-
	M-RYGB	2 (0.22%)	
	SADI-S	1 (0.11%)	
Patient’s risk factors, n (%)	Arterial hypertension	3 (0.33%)	0.162***
	Diabetes Mellitus	2 (0.22%)	0.157***
	Hyperlipidemia	2 (0.22%)	0.585***
	Smoking habits	1 (0.11%)	0.773***
	OSA	1 (0.11%)	0.465***
	Combined oral contraceptive use	1 (0.11%)	0.616***
	Reintervention	1 (0.11%)	0.06**
Mortality, n (%)		0 (0%)	-

None of these patients had previous history of thromboembolic events, chronic venous insufficiency, or other diseases that could increase the risk of VTE. Furthermore, there were 4.9% of hemorrhagic complications. The median length of hospital stay was two days. No mortality reported (Table 6).

**Table 6:** VTE and hemorrhagic complications and postoperative outcomes. VTE: venous thromboembolic; DVT: deep venous thrombosis; PE: pulmonary embolism; ICU: intensive care unit; IQR: interquartile range; N/A: not applicable

Variables	n (%)	Management
VTE complication, n (%)	4 (0.44%)	-
a. DVT	2 (0.22%)	Anticoagulation
b. PE	1 (0.11%)	ICU admission
c. DVT+PE	1 (0.11%)	Hospital readmission
Hemorrhagic complication, n (%)	44 (4.9%)	-
a. Abdominal wall hematoma	9 (1%)	Conservative management
b. Gastrointestinal bleeding (melena/hematochezia)	28 (3.1%)	3 requiring blood transfusion and 1 requiring reintervention
c. Hemoperitoneum	7 (0.8%)	Reintervention
Length of stay, days (IQR)	2 (2-3)	N/A
Mortality, n (%)	0 (0%)	N/A

## Discussion

Thromboembolic complications represent a main cause of morbidity and mortality following BS and are an important concern with clinical and economic consequences. However, there is still no consensus regarding the optimal VTE screening method, chemoprophylactic agent, and prophylaxis duration.

All bariatric surgery patients are at least at moderate to elevated risk of VTE, therefore thromboprophylaxis is recommended [13,14]. During these three years period, we registered 0.22% of DVT, 0.11% of PE, and 0.11% of both in a single patient. Considering the occurrence of VTE 30 days of the index BS, we only had one VTE complication (0.1%). Our rate of thromboembolic complications is the lowest reported so far [3-5]. In a large study including more than 90.000 patients who underwent BS between 2007 and 2012, the prevalence of thirty-day total VTE events was 0.39% and the mortality rate 0.1%, with a 30-day total frequency of DVT and PE of 0.22% and 0.17%, respectively [15].

Thromboembolic complications are an important cause of morbidity after BS increases health care costs. Preoperative identification of patients at higher risk will allow for screening, prophylaxis, and prevention in the bariatric surgery population. The postoperative VTE risk factors among these patients can be categorized as patient-related and procedure-related. Thus, male gender, advanced age, preoperative patient weight and BMI, smoking status, congestive heart failure, hypertension, hormonal therapy, immobility, hypercoagulable condition, chronic obstructive pulmonary disease, and prior history of VTE are some of the patient-related risk factors for VTE [6,16,17]. The procedure-related factors include long operative time, bariatric procedure type, and postoperative complications [16,17]. Bariatric studies also reported an increased risk in open procedures [3,16]. We report only laparoscopic surgeries, which could contribute to our results.

A tool that can assess the VTE risk including specific risk factors to the population undergoing BS is warranted. Aminian et al. using a large multicenter database proposed a risk calculator for post-discharge VTE events in patients undergoing BS, based on previously described risk factors [15]. Finks et al. used the Michigan Bariatric Surgery Collaborative database to create a risk calculator for all postoperative VTE events post-BS [18]. Nonetheless, none has been globally accepted and implemented in daily clinical practice.

With shorter hospital stays, VTE has become more common in the post-discharge setting. One consistent finding is that most VTE events occur within the first 30 days after hospital discharge [11,16,19-22]. Helm et al. reported a postoperative 0.5% VTE incidence with an average time of 14.3 days to DVT diagnosis and 12.1 days to PE. In this study, 80% of VTE events occurred after hospital discharge [17].

In our practice, all patients are discharged with recommendations to maintain walking activities, judicious hydration, and chemical thromboprophylaxis. The PE was the only thrombotic event that occurred while the patient was still hospitalized, on day 12. This woman was a high-risk patient as she had an intestinal perforation with peritonitis requiring reoperation. This also corroborates the study of Helm et al. reporting that VTE likelihood was directly related to postoperative complications with 22.6% of the patients who developed a VTE experiencing first a major complication [17]. All the other thromboembolic events happened after patients’ discharge - two DVTs occurred nine and 16 months after surgery, respectively, and combined DVT and PE were verified after two months. One can argue that the PE which occurred 12 days after surgery was the only thromboembolic event worth mentioning. Moreover, we describe 44 hemorrhagic complications - nine abdominal wall hematomas, 28 gastrointestinal bleeding (three requiring blood transfusion and one reintervention for anastomosis reinforcement), and seven hemoperitoneum.

More complex procedures are associated with higher risk of VTE, probably because these surgeries are associated with longer operating time and higher preoperative BMI. Chan et al. identified operative time >3 h as an independent predictor of postoperative VTE and the preoperative BMI as an independent predictor of operative time [23]. A confounding factor is the characteristics of patients undergoing more complex procedures, who generally have a higher BMI or other comorbidities, which may themselves influence the risk of VTE. We performed 901 bariatric surgeries, 95.2% primary and 4.8% revisional. There were no VTE events among patients undergoing revisional bariatric surgery.

Our results must be seen in a context of an experienced and high‑volume bariatric center - the short duration of surgery with a dedicated bariatric surgical team, the laparoscopic approach in 100% of procedures, early ambulation on the same day of the surgery, early discharge, specialized anesthesia care with optimal analgesia, and a short recovery time all contribute for the satisfactory overall results.

All patients are evaluated during the preoperative anesthesia consultation for OSA using STOP-Bang questionnaire - a validated screening tool for OSA in surgical patients (Table 7) [24]. The immediate postoperative non-invasive ventilatory approach is guided according to this evaluation (Table 8).

**Table 7:** STOP-BANG score for OSA diagnosis before surgery. STOP-Bang scoring model adapted from Chung et al. (2008) (CC-BY-NC) [24]. Adapted with permission from Wolters Kluwer Health, Inc.

STOP-Bang scoring model					
1. Snoring: Do you snore loudly (loud enough to be heard through closed doors)?					
Yes					
2. Tired: Do you often feel tired, fatigued, or sleepy during daytime?					
Yes					
3. Observed: Has anyone observed you stop breathing during your sleep?					
Yes					
4. Blood Pressure: Do you have or are you being treated for high blood pressure?					
Yes					
5. BMI: BMI more than 35 kg/m^2^?					
Yes					
6. Age: Age over 50 years old?					
Yes					
7. Neck circumference: Neck circumference >40 cm?					
Yes					
8. Gender: Male?					
Yes					
High risk of OSA: answering yes to three or more items					
Low risk of OSA: answering yes to less than three items					

**Table 8:** Non-invasive ventilation in the postoperative period protocol according to STOP-Bang questionnaire. OSA: obstructive sleep apnea; CPAP: continuous positive airway pressure; BiPAP: bilevel positive airway pressure Table is adapted from Chung et al. (2008) (CC-BY-NC) [24]. Adapted with permission from Wolters Kluwer Health, Inc.

STOP-Bang questionnaire	Non-invasive ventilation
STOP-Bang 0-2	Nasal low-flow cannula
STOP-Bang 3-4	Nasal low-flow cannula prophylactic CPAP
STOP-Bang >5 BMI >50 kg/m^2^ peripheral oxygen saturation (SpO_2_) >90% with supplementary oxygen	Therapeutic CPAP in early postoperative
Obesity with OSA using domiciliary CPAP	Therapeutic CPAP in early postoperative
Obesity-hypoventilation syndrome	Prophylactic BiPAP

All patients with BMI >50 kg/m^2^, with OSA, or at high risk of adverse cardiovascular or respiratory events are admitted to the intensive care unit for postoperative care. This ventilatory strategy aims to prevent postoperative pulmonary atelectasis and consequent respiratory complications such as pneumonia or respiratory failure, which could delay ambulation, prolong hospital length of stay and lead to complications including VTE events.

Although slightly reported in the literature, we believe that this active ventilatory approach in the immediate postoperative period, combined with the short operative time, non-opioid-based pain control methods, and the rapid onset of ambulation, may help to explain the reduced rate of respiratory complications and, consequently, PE recorded in our population.

LMWH is a hydrophilic drug and largely remains in the intravascular compartment. Patients with obesity may have disproportionately more adipose tissue, causing an overdose and bleeding when they are treated with LMWH adjustable for their total body weight, supporting the idea that obese patients may not need a higher dose of enoxaparin as we thought [25,26]. Recent position papers continue to recommend routine prophylactic measures to prevent VTE, which includes both DVT and PE, after bariatric surgery [9,16,27].

The ERAS Society recommends mechanical and pharmacological measures with an individualized dosage of LMWH. The first dose should be given 8-12 h postoperatively during three to four weeks, having no data supporting a twice-a-day prophylaxis [14]. Clinic Practice Guidelines of the European Association for Endoscopic Surgery on bariatric surgery state that no recommendation can be made regarding dose and duration of pharmacological prophylaxis [27].

A joint group including the American Society for Metabolic and Bariatric Surgery recommends a prophylactic regimen that includes compression devices and LMWH given 24 h after surgery. Also extended chemoprophylaxis after hospital discharge for high-risk patients [9].

Strategies to reduce the risk of VTE include early postoperative mobilization; mechanical compression devices, such as intermittent pneumatic compression or graduated compression stockings; anticoagulant drugs; and vena cava filters. Although recommended by most societies and guidelines, there is currently limited evidence showing a reduction in the incidence of fatal embolism with the use of mechanical methods [28]. Patient compliance and correct use of socks are two frequently mentioned problems. Nevertheless, an important advantage of mechanical thromboprophylaxis is the lack of bleeding potential, which is the reason why it is recommended in patients with high bleeding risk [29].

For all our patients, we start chemical thromboprophylaxis 12 h after surgery at a dosage of enoxaparin 40 mg once daily, and for patients with BMI ≥50 kg/m^2^ or weight ≥150 kg, 60 mg once daily. All patients prolong this treatment for three weeks. In high-risk patients according to Caprini score (Table 9), the management also includes mechanical compression during and after surgery until ambulation. In patients requiring ICU admission, intermittent pneumatic compression is used. All patients start an early ambulation and hydration routine. In patients with risk of bleeding, mechanical methods of thromboprophylaxis are primarily used. In patients with very high risk of VTE event, an association of mechanical and pharmacological methods is applied.

**Table 9:** Caprini risk score assessment for VTE. The table (Caprini risk assessment model, version 2013) is adapted from Cronin et al. [13].

1 point	2 points	3 points	5 points
Age 41-60 years	Age 61-74 years	Age 75 or over	Elective major lower extremity arthroplasty
Minor surgery planned (<45 min)	Current or past malignancies (excluding skin cancer but not melanoma)	History of deep vein thrombosis or pulmonary embolism	Hip, pelvis, or leg fracture
Past major surgery (>45 min) within the last month	Planned open or laparoscopic major surgery (>45 min) and arthroscopic surgery	Family history of thrombosis	Multiple trauma
Visible varicose veins	Non-removable plaster cast in the lower limb (<1 month)	Personal or family history of thrombosis	Acute spinal cord injury causing paralysis
Swollen legs (current)	Central venous access (<1 month)		Stroke
Overweight or obese (BMI ≥25 kg/m^2^)	Confined to a bed for 72 h or more	Caprini risk category based on total risk score	
History of inflammatory bowel disease	1 point for women only	Total score	Category
Myocardial infarction	Current use of birth control or hormone replacement therapy	0-2	Low
Congestive heart failure (<1 month)	Pregnant or had a baby within the last month	3-4	Moderate
Sepsis (<1 month)		5-6	High
Lung disease	History of unexplained stillborn infant, recurrent spontaneous abortion (more than 3), or premature birth	7-8	Very high
Medical patient is currently on bed rest		>9	Highest

There is still no consensus regarding the correct dose of LMWH, weight-adjusted or standard dose. Some bariatric centers currently apply protocols with adjustable doses expressing concerns with thromboembolic events, reporting, on the other hand, more hemorrhagic complications, that are, itself, a risk factor. Despite recommendations to use an increased dose of LMWH in bariatric patients according to their weight, in population-based studies, the rate of VTE after laparoscopic bariatric surgery seems to be relatively low with standard regimens for antithrombotic prophylaxis [2]. The incidence of major bleeding seems to increase using weight-adjusted doses of heparin with no advantage in terms of VTE reduction. As we found in our experience, the incidence of VTE events was low with standard dose of LMWH, supporting these findings.

Limitations

The present study is primarily limited by its retrospective nature. The diagnosis of VTE events has not been routinely screened in all patients, just in the presence of clinical symptoms.

## Conclusions

The lack of consensus and the difficulty to achieve a universal VTE prophylaxis approach may be linked to the side effect of thromboprophylaxis that bothers the surgeon, the bleeding. We reported a lower rate of thromboembolic events than previously reported in literature using this standardized thromboprophylaxis protocol for bariatric patients. We believe that not only the pharmacological measures but also the non-pharmacological ones are the key for our good results. We propose that this protocol could be implemented in other bariatric centers as it is associated with reduced morbidity and mortality.
